# UAV Image-Based Crop Growth Analysis of 3D-Reconstructed Crop Canopies

**DOI:** 10.3390/plants11202691

**Published:** 2022-10-12

**Authors:** Karsten M. E. Nielsen, Hema S. N. Duddu, Kirstin E. Bett, Steve J. Shirtliffe

**Affiliations:** 1 Department of Plant Sciences, College of Agriculture and Bioresources, University of Saskatchewan, Saskatoon, SK S7N 5A8, Canada; 2 Agriculture Agri-Food Canada, 107 Science Place, Saskatoon, SK S7N 0X2, Canada

**Keywords:** breeding efficiency, digital plant volume, high-throughput, plant growth rate, plant phenotyping

## Abstract

Plant growth rate is an essential phenotypic parameter for quantifying potential crop productivity. Under field conditions, manual measurement of plant growth rate is less accurate in most cases. Image-based high-throughput platforms offer great potential for rapid, non-destructive, and objective estimation of plant growth parameters. The aim of this study was to assess the potential for quantifying plant growth rate using UAV-based (unoccupied aerial vehicle) imagery collected multiple times throughout the growing season. In this study, six diverse lines of lentils were grown in three replicates of 1 m^2^ microplots with six biomass collection time-points throughout the growing season over five site-years. Aerial imagery was collected simultaneously with each manual measurement of the above-ground biomass time-point and was used to produce two-dimensional orthomosaics and three-dimensional point clouds. Non-linear logistic models were fit to multiple data collection points throughout the growing season. Overall, remotely detected vegetation area and crop volume were found to produce trends comparable to the accumulation of dry weight biomass throughout the growing season. The growth rate and G50 (days to 50% of maximum growth) parameters of the model effectively quantified lentil growth rate indicating significant potential for image-based tools to be used in plant breeding programs. Comparing image-based groundcover and vegetation volume estimates with manually measured above-ground biomass suggested strong correlations. Vegetation area measured from a UAV has utility in quantifying lentil biomass and is indicative of leaf area early in the growing season. For mid- to late-season biomass estimation, plot volume was determined to be a better estimator. Apart from traditional traits, the estimation and analysis of plant parameters not typically collected in traditional breeding programs are possible with image-based methods, and this can create new opportunities to improve breeding efficiency mainly by offering new phenotypes and affecting selection intensity.

## 1. Introduction

Plant growth rate is an essential component of plant fitness, as rapid growth rate will increase the ability of the crop to compete with other species and capture sunlight, carbon dioxide, and water efficiently. As above-ground plant material is responsible for sunlight harvesting and gas exchange processes, the quantification of above-ground plant material can give insight into the production potential of various crop species [1].

Evaluation of plant growth rate is typically based on either height [2,3,4] or biomass [5]. The collection and measurement of biomass are tedious and time-consuming, resulting in a significant expense in large plant research and breeding programs. More importantly, biomass evaluation in a field setting is inherently destructive necessitating larger areas and more resources [5]. While biomass measurements and growth evaluation may be made more easily in indoor, potted environments, plants are unlikely to perform equally to field-based experiments, potentially reducing the utility of results [6]. Cereal crops such as corn and wheat tend to have a height that is highly correlated with biomass [7]. Because of this, simply measuring height can give a very good estimate of biomass. Therefore, by measuring the rate of height increase over a period of time, the growth rate may be effectively deduced [2,4]. However, in bushy crops such as lentil, biomass is less highly correlated with height, and the ability to predict biomass based on height alone is reduced. When all plants observed are of the same species and variety and are expected to grow and develop similarly, models may be produced to adjust for variation resulting from lateral branching in the horizontal plane [8]. In cases where the precise morphology of plants is unknown or when evaluating diverse populations, biomass estimation from height alone would likely result in insufficient accuracy and reproducibility.

The collection of multiple overlapping images with high spatial and temporal resolution is possible with UAVs [9]. Using SfM (structure from motion) techniques, the aerial imagery can be used to produce high-density 3-D point clouds, from which several plant growth parameters can be measured. This approach has been used previously to measure grassland [10] and forest/shrub biomass [11,12,13]. Similar work has used 3-D laser scanners to produce 3-D point clouds for analysis [14,15]. While previously published studies use SfM techniques to acquire height information [16,17,18] and the height information used to calculate plant growth rate [19,20], little to no literature was available evaluating the SfM-derived crop volume of field crops in a field environment.

Above-ground vegetation biomass, area, and volume are reasonably well-correlated and may be used to obtain generalized inferences at various times in the growing season. Further analysis has the potential to evaluate parameters of biomass, area, and volume basis over time and across different environments. The objective of this experiment was to assess the potential for quantifying plant growth rate using UAV-based imagery collected multiple times throughout the growing season. Rapid, non-destructive evaluation of plant growth rate could be used in various plant research and breeding programs to efficiently evaluate material best suited for particular environments and stresses in a field environment. Furthermore, a similar methodology may find utility in larger-scale operations such as predicting crop yield by producers, crop insurance agencies, and for yield monitoring [21].

## 2. Results and Discussion

### 2.1. Ground-Measured Data

As each genotype examined expressed a large variation in maturity; earlier-maturing genotypes were well past physiological maturity and had completed senescence before the last harvest date. Therefore, the fitted three-parameter non-linear logistic curve was applied to a dataset truncated to omit any end-of-season decline in dry weight biomass.

**Figure 1:**
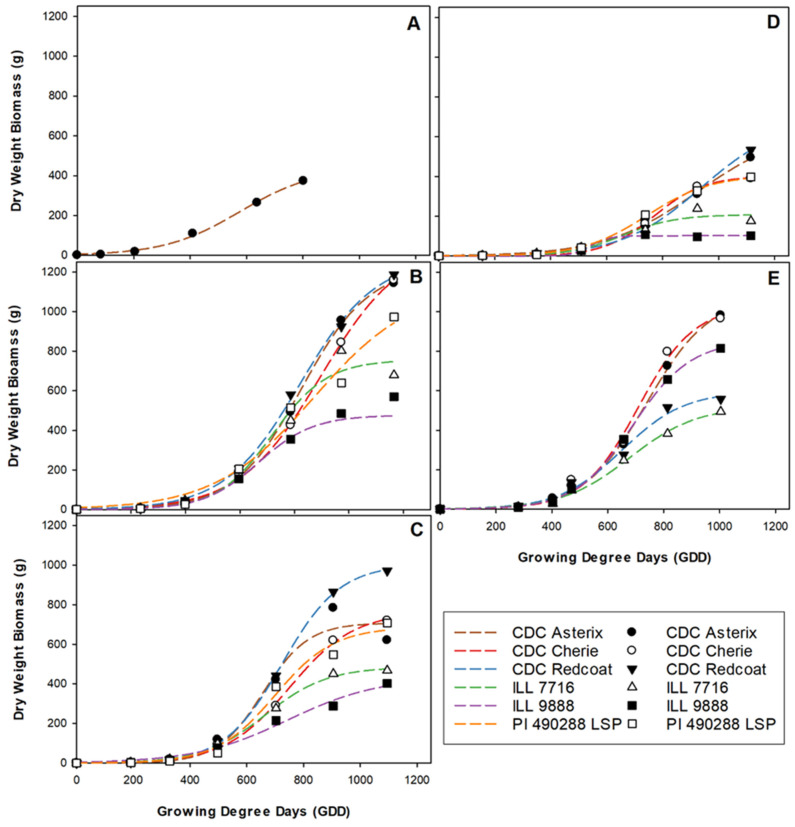
Three-parameter growth curves showing dry weight biomass accumulation for each genotype throughout the growing season at each site-year; Nasser 2017 (**A**), Rosthern 2017 (**B**), Sutherland 2017 (**C**), Nasser 2018 (**D**), and Rosthern 2018 (**E**). Data at Nasser 2017 was best described by combining all genotypes within the model.

**Figure 2:**
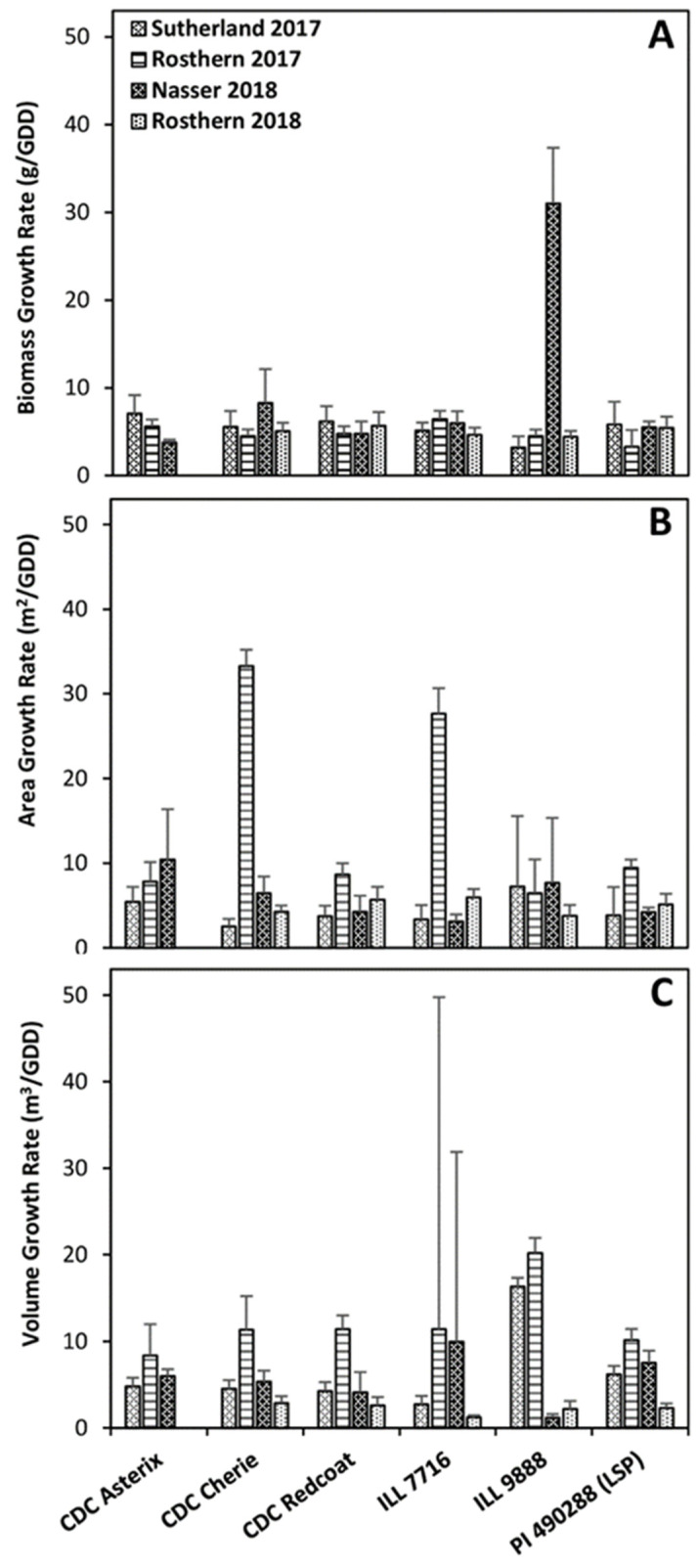
Estimated growth rate parameters: for dry weight biomass (**A**), vegetation area (**B**), and plot volume (**C**) are displayed for each genotype at each site-year. Error bars show standard error.

**Figure 3:**
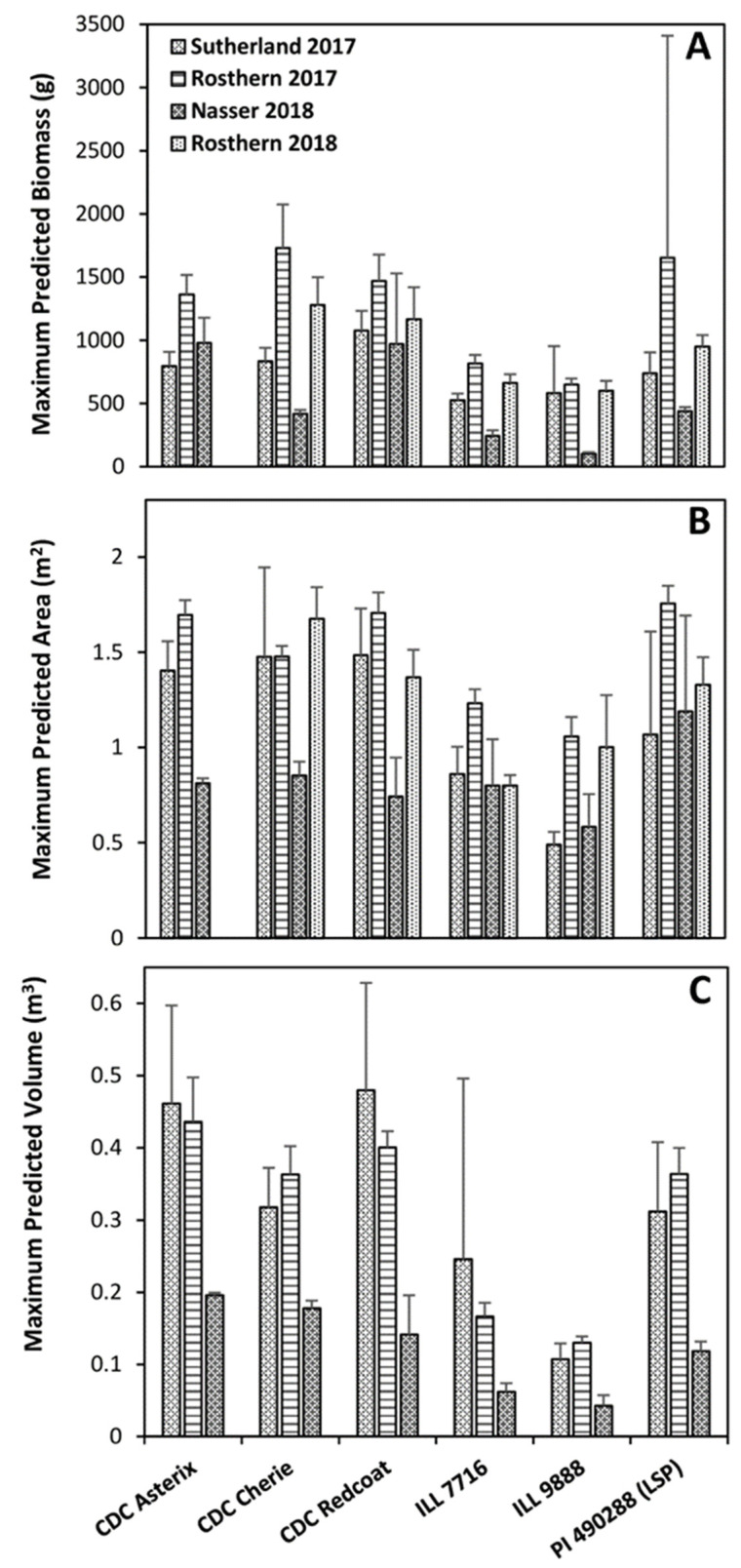
Estimated maximum predicted growth parameters: for dry weight biomass (**A**), vegetation area (**B**), and plot volume (**C**) of each genotype at each site-year. Error bars show standard error.

The average dry weight biomass varied substantially among site-years, with most genotypes in Rosthern 2017 experiencing nearly three times the dry weight biomass of those in Nasser 2018. While 1100 to 1200 GDD was calculated at most site-years, it is noteworthy that the Nasser 2017 trial was seeded later in the spring and therefore was only exposed to approximately 800 GDD. It can be assumed that this reduced thermal growth period likely influenced the total accumulated biomass. Despite the variability among the site-years, the genotypes were true to their biomass accumulation class as expected (Figure 1). In most site-years, CDC Redcoat and CDC Asterix were the two largest genotypes, and ILL 9888 and ILL 7716 were the smallest based on the predicted estimates (Figure 1, Figure 2 and Figure 3). Although the biomass accumulation for Nasser 2017 was described by combining all the genotypes, the trends for the individual genotypes followed other site-years (data not shown).

### 2.2. Two-Dimensional Analysis

**Figure 4:**
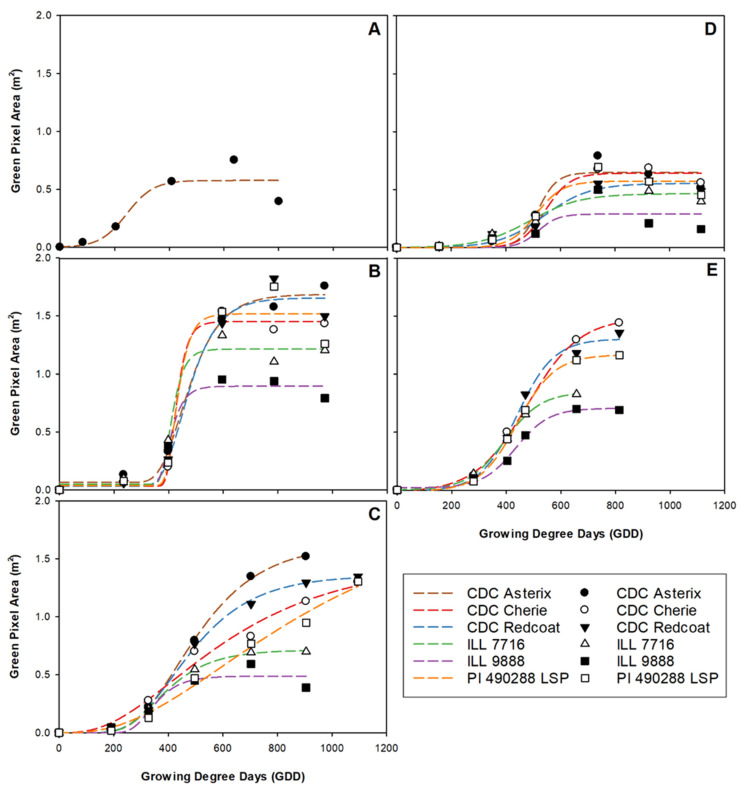
Three-parameter growth curves showing green pixel area accumulation for each genotype throughout the growing season at each site-year; Nasser 2017 (**A**), Rosthern 2017 (**B**), Sutherland 2017 (**C**), Nasser 2018 (**D**), and Rosthern 2018 (**E**). Data at Nasser 2017 was best described by combing all genotypes within the model.

Accumulation and maximum green pixel area values often differed among genotypes and site-years (Figure 4). To some extent, image-based green pixel area trends followed that of manual-collected dry weight biomass. A low overall dry weight biomass and green pixel area were observed in Nasser 2017 and 2018. Similarly, a high overall dry weight biomass and green pixel area was reported at the Rosthern location in both years.

However, the order of green pixel area for individual genotypes did not match the dry weight biomass. For example, in three out of five site-years, CDC Cherie produced a higher green pixel area than CDC Redcoat, a genotype that had higher dry weight biomass. This relatively poor association between manually collected biomass and the digital approach is partly due to the accumulation of height and density that cannot be accounted for using a 2-D approach that measures only groundcover. It should be noted that the 2-D approach, in most cases, identified large, medium, and small genotypes correctly, with CDC Asterix, CDC Cherie, and Redcoat generally having the greatest area and ILL 9888 usually having the lowest area (Figure 4).

**Figure 5:**
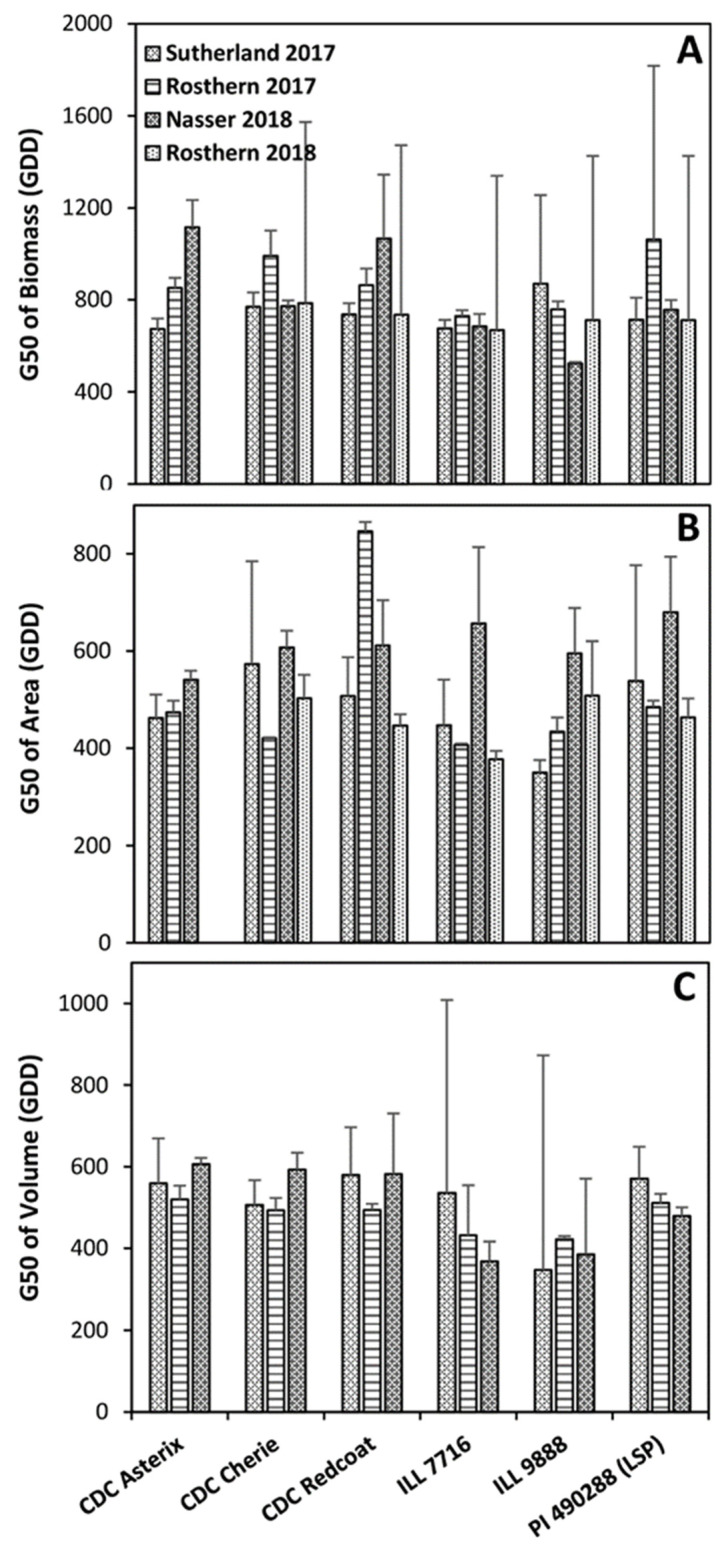
Estimate of G50: for dry weight biomass (**A**), vegetation area (**B**), and plot volume (**C**) of each genotype at each site-year. Error bars show standard error.

The values of G50, or the time required to reach 50% of total growth, suggest a significant variation among both genotype and environment (Figure 5). Differences in seeding time and weather conditions are probably responsible for significantly different G50 values across site-years. Additionally, G × E interactions occurred, which caused certain genotypes to be differentially suited to specific environments. Overall, the G50 of the green pixel area is significantly lower than the G50 of biomass (Figure 5). This indicates that the crop reaches 50% of the total ground cover earlier in the season than 50% of total biomass. Because rapid groundcover increases the competitive ability and resource-capturing capability, this trait might greatly benefit breeding programs and other crop research initiatives [22,23].

Green pixel area growth rate values differ significantly among several genotypes and site-years. At Rosthern 2017, CDC Cherie and ILL 7716 had considerably larger green pixel area growth rates than other genotypes in the same site-year as well as all other genotypes at all other site-years (Figure 2). These large variations in green pixel area growth rate are likely indicative of the ideal growing conditions experienced in Rosthern 2017 and the ability of CDC Cherie and ILL 7716 to rapidly produce groundcover under such growing conditions.

Rapid groundcover and seedling vigor are essential for the crop to effectively establish and reduce the producers’ need for weed control [23]. As such, groundcover and vigor measurements are often obtained manually in crop breeding programs due to their strong relationships with interspecific competitive capabilities, tolerance to biotic and abiotic stresses, and final seed yield [22,24,25]. Similar studies in maize using spectral indices for biomass estimation have been performed, attaining a high correlation with plant biomass and a high level of repeatability [26,27].

### 2.3. Three-Dimensional Analysis

**Figure 6:**
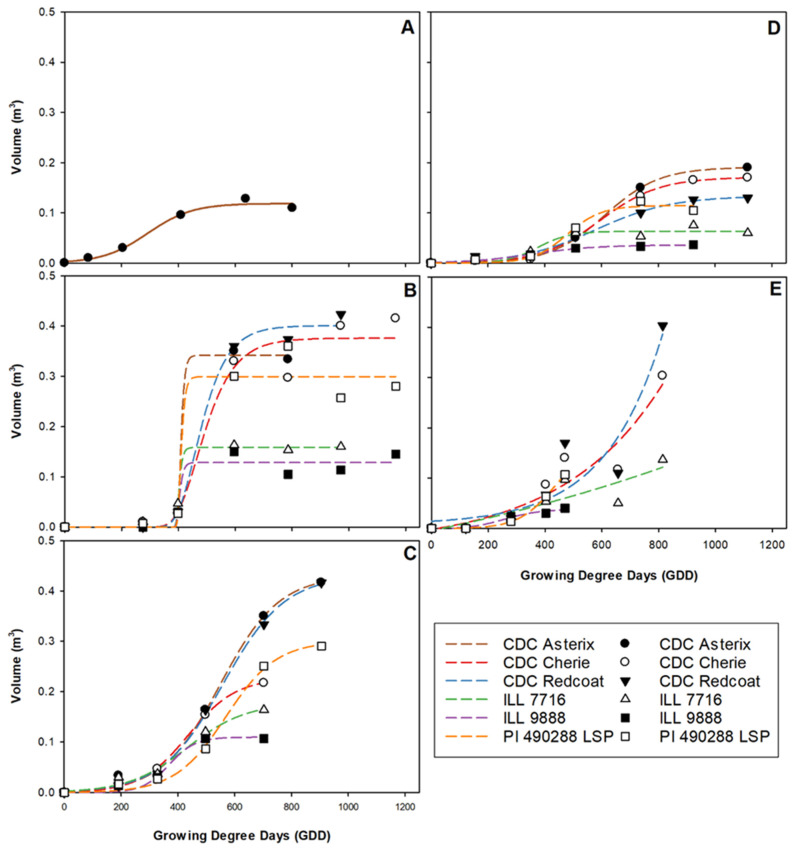
Three-parameter growth curves showing volume accumulation for each genotype throughout the growing season at each site-year; Nasser 2017 (**A**), Rosthern 2017 (**B**), Sutherland 2017 (**C**), Nasser 2018 (**D**), and Rosthern 2018 (**E**). Data at Nasser 2017 was best described by combing all genotypes within the model.

Large differences in volume accumulation were observed among genotypes and site-years (Figure 6). Overall, volume growth trends follow a pattern that appears more similar to those observed for dry weight biomass than did green pixel area (Figure 1, Figure 4 and Figure 6). Genotypes fell in a very similar order at most site-years for dry weight biomass and volume (Figure 1 and Figure 6) and the model output maximum predicted growth (Figure 3). Overall, CDC Asterix, CDC Redcoat, and CDC Cherie tended to have greater maximum predicted growth. In contrast, ILL 9888 consistently had the lowest maximum predicted growth, closely followed by ILL 7716. G50 values were relatively similar both among genotypes and among site-years.

The volume data obtained at Rosthern 2018 was poorly described using the curve chosen for data analysis in this experiment (Figure 6E). This is likely due to the relatively late seeding date followed by ideal growing conditions throughout the season, culminating in a rapid maturation rate due to the sudden disease onset. Between the time of the greatest plot volume and the next image collection date, volume had declined significantly, and data was truncated to avoid false late-season underestimation by the model. When fitted to a three-parameter logistic curve, this resulted in a poor fit of the growth curve, resulting in an unrealistically large maximum predicted volume and G50 estimations and unrealistically small volume growth rate, or rate of plot volume increase estimations [28]. Therefore, the maximum predicted volume and G50 values of Rosthern 2018 were not considered for the analysis (Figure 3C and Figure 5C).

Volume growth rate determines the volume increase over time, and it can be used to describe the rate of 3-D space-filling capability of an accession. Similar to area growth rate, however, it is noteworthy that many genotypes experienced relatively high volume growth rates in Rosthern 2017. This indicates good growing conditions leading to a rapid growth rate. In contrast, such high growth rates were not observed in the case of dry weight biomass in spite of high overall dry weights reported for most genotypes in 2017.

Together with G50 of volume, the volume growth rate has potential utility in plant breeding programs for germplasm selection. For instance, these traits can be used to identify germplasm with greater and more rapid early-season growth. Plants that fill space earlier in their lifecycle will have a competitive advantage over weeds and greater resource-harvesting capability. A greater ability to collect resources such as sunlight, oxygen, water, and nutrients early in the growing season will prolong the time resources are collected in large amounts, thereby increasing yield potential [29]. Additionally, greater growth and biomass accumulation in times of stress are generally beneficial, and rapid early-season growth may aid in stress tolerance [30,31].

As the trends shown in Figure 6 are based on the maximum observed value before data truncation, this approach is still believed to be useful in estimating plant size and growth parameters. A lack-of-fit test was performed for all site-years and was insignificant for Rosthern 2018, indicating that the model fit the data to an acceptable level, so parameter outputs were considered useful for relative comparison among genotypes within the Rosthern 2018 site-year. For this experiment, parameter outputs for Rosthern 2018 were not considered directly comparable to other site-years. Trends based on the maximum measured values for each trait before data truncation were used for environmental and varietal comparison of Rosthern 2018 instead (Figure 3).

As standard errors for G50 were quite large at several site-years for all traits examined, it is difficult to make conclusive comparisons (Figure 5). However, ILL 7716 and ILL 9888 did tend to have lower G50 values than did CDC Asterix, CDC Cherie, and CDC Redcoat. Overall, G50 is much lower for volume than biomass (Figure 5A,C). This indicates that 50% of the volume was reached earlier than 50% of biomass was accumulated. As space-filling ability has essential effects on competition and resource harvesting, this may be a valuable trait to consider for variety development.

G50 and GDD required to reach the maximum predicted growth might be more useful parameters for germplasm selection in breeding programs than growth rate at the early stages of plant development, as the growth rate is not expected to vary significantly until the critical density of each accession has been reached [32]. The critical density of a crop may be estimated using G50 values from remotely evaluated vegetation area or plot volume [32]. They also suggested two models that can be used to determine the critical density of a crop: a biophysical model, which considers plant radius in relation to neighboring plants, and a metabolic model, which assumes all plants have a set metabolic rate, which is collectively maximized in a given area when plants are densely packed. They identified a similar initial growth rate in a variety of crops. Once the critical density was reached, however, growth slowed or stopped, with the growth of individual plants only occurring when resources were made available by the mortality of neighboring plants. Therefore, the prediction of critical density based on G50 could be of value to crop research programs with an interest in intraspecific competition or seeding density [33], among other topics. Vegetation area and volume parameters may also be useful in predicting the critical density of individual genotypes, giving utility in evaluating the interspecific and intraspecific competitive capabilities of individual genotypes.

### 2.4. Vegetation Area as a Measurement of Plot Biomass

The dry weight biomass was highly correlated with green pixel area at many data collection timings throughout the experiment (Table 1). In a study by Tomasel et al. [34], the foliar area of bunchgrass was evaluated using a chromaticity-based pixel counting method that utilized individual RGB images collected manually from 1.4 m above ground level. The study found a highly significant correlation between green pixels and dry weight biomass in field situations without a large number of overlapping leaves. Therefore, green pixel area is expected to be a useful parameter to estimate lentil biomass early in the growing season before significant vertical growth occurs.

At the first biomass sample timing, most locations had a high correlation between biomass and green pixel area (>0.84). Interestingly, the correlation between volume and area at the first data collection timing is low at all site years, suggesting that area is a more useful parameter for early-season measurement of plant growth. At collection 3 in Nasser 2017, collections 4 and 5 in Rosthern 2017 and Rosthern 2018, and collection 5 in Sutherland 2017 and Nasser 2018, the correlation between biomass and area experienced a decrease from the trend. Because this mid-season dip in correlation occurred at every trial location, it seems unlikely to be caused by an error in data collection. The timing of the decrease coincided with the period of the greatest rate of biomass accumulation (Figure 1) so it seems likely that the dip in correlation was due to an increase in plant height that was not accompanied by an increase in the plant area. As the season further progressed, plant stems became unable to support the increasing weight of the canopy, and lodging occurred. Additionally, leaf and tendril growth was expected to continue in some genotypes after the increase in canopy height slowed [35]. Collectively, this led to the infilling of inter-row space with vegetation and again increased the correlation between plant biomass and remotely measured plant area. Using a similar methodology in dry bean, Sankaran et al. [36] noted an increasing correlation between area and biomass from early to mid-season data collection but significantly reduced correlation during late pod-fill. A similar reduced late-season correlation only occurred at one location, Nasser 2017, likely due to a high level of disease late in the growing season that resulted in rapid senescence.

### 2.5. Vegetation Volume as a Measurement of Plot Biomass

Compared to the green pixel area, volume correlation with dry biomass followed a generally increasing trend at all locations except Rosthern 2017 throughout the growing season (Table 1). Volume was poorly correlated with biomass at the first biomass sampling time at all trial locations, with the highest correlation being 0.36 at Sutherland 2017. This low early-season correlation was likely due to insufficient resolution to produce a meaningful volume estimation on the small plant sizes observed. On the second biomass sampling date, the correlation between biomass and volume was reasonably high (>0.80) at all locations except Rosthern 2018, with a correlation of 0.43, and Nasser 2018, with a correlation of 0.42. For the remainder of the growing season, the correlation between biomass and volume remained relatively consistent or slightly increased when observed over time. These results are consistent with SfM-based biomass estimation in various herbaceous crops, including *Vicia sativa*, *Triticum sativum*, *Secale cereale*, *Medicago sativa*, and *Triticale* performed by Gil-Docampo et al. [37], which concluded that SfM to be a potential tool for biomass estimation in field crops. Sun et al. [38] found a coefficient of determination value of 0.98 when comparing LiDAR point cloud-derived volume data of man-made models of cotton plants with a manually measured volume of the model.

Dixit et al. [39] identified a large variation in harvest index (HI) and biomass in lentil varieties and indicated a need to select both high HI and high biomass varieties to identify high-yielding germplasm. Using late-season plot volume measurements combined with predicted or manually measured seed yield, both above-ground crop biomass and HI parameters may be calculated to allow the selection of material with high-yield potential for further analysis and breeding development uses. Currently, evaluation of HI is highly uncommon in plant breeding endeavors due to the enormous time required to measure above-ground biomass despite being a highly informative trait for developing efficient crop varieties. 

## 3. Materials and Methods

### 3.1. Germplasm

Six diverse lentil genotypes—CDC Asterix, CDC Cherie, CDC Redcoat, ILL 7716, ILL 9888, and PI 490288 LSP, were selected for biomass and high-throughput phenotyping analysis from a larger lentil diversity panel (LDP) grown in previous seasons [40]. The subset was selected specifically based on the observed diversity of biomass, canopy height, plant architectural traits, and ground cover. In Rosthern 2018, CDC Asterix was omitted, and only five genotypes were analyzed due to an error during seeding.

### 3.2. Experimental Design

**Figure 7:**
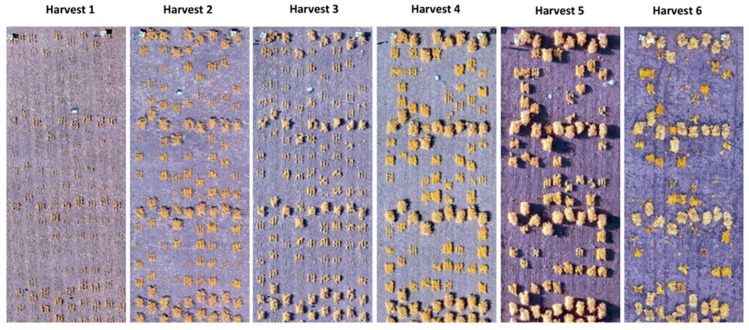
Six orthomosaics represent six biomass harvest time points. Whole-plot biomass was measured approximately every two weeks throughout the growing season (harvests 1 to 6), with aerial images collected within 24 h prior to destructive sampling. Six diverse lentil genotypes were grown in a Randomized Complete Block Design (RCBD) with three replicates in five site years. Larger plots in each orthomosaic are pea plots used to separate replicates.

The trial was arranged in a Randomized Complete Block Design (RCBD) with three replicates separated by a single range of pea plots (Figure 7). Each microplot was seeded at a rate of 70 seeds per 1 m^2^ plot planted in three rows. Five site-years were observed, with Nasser (52°09′ N, 106°31′ W) and Rosthern (52°41′ N, 106°17′ W) locations in both 2017 and 2018, and an additional location of Sutherland (52°10′ N, 106°30′ W) in 2017. Both Nasser and Sutherland locations had a Dark Brown Chernozemic clay soil with a pH of 7.2, while Rosthern had a Black Chernozemic loam with a pH of 7.2. Throughout the crop growth period (May to August), the mean temperature ranged from 11 to 20 °C with total precipitation varied between 132–166 mm for all locations and years. Weeds were manually controlled to ensure weed-free imagery and biomass samples and to eliminate interspecific competition. Six plots of each accession were established per replication to allow whole-plot biomass analysis approximately once every two weeks throughout the growing season (Figure 7).

### 3.3. Field Data Collection

Whole-plot biomass was measured by cutting all the above-ground material at ground-level approximately bi-weekly. Plants were collected within 24 h before overhead images were captured. Samples were dried at 71 °C for 72 h or until oven moisture was below 2%. Dried samples were weighed immediately following removal from the drying oven. Growing degree days (GDD) were calculated at each trial location using hourly recorded temperature data collected from respective trial locations. The equation used to describe GDD was:

(1) $$Accumulated\;GDD=\sum [\left(\frac{T_{MAX}+T_{MIN}}{2}\right)-T_{BASE}]$$

where $T_{MAX}$ is the maximum recorded daily temperature, $T_{MIN}$ is the minimum recorded daily temperature, and $T_{BASE}$ is assumed minimum temperature where growth occurs. For this experiment, $T_{BASE}$ of lentil was assumed to be 5 °C [41,42]. If $T_{MAX}$ or $T_{MIN}$ were less than $T_{BASE}$, they were considered to be zero as described by McMaster and Wilhelm [43]. GDD accumulation was considered to begin on the date of seeding.

### 3.4. Aerial Image Acquisition

UAVs were used as rapid overhead image collection platforms. Two Draganfly UAVs (Draganfly Innovations, Saskatoon, SK, Canada)—one Draganflyer X4-P model, and one Draganflyer Commander model, were used interchangeably to collect images. Both UAVs were quadcopters outfitted with a gimbal-stabilized camera mount designed to accept various cameras.

Most images were collected using either a modified consumer-grade 24.3 MP Sony α5100 series camera (Sony Corporation, Minato, Tokyo, Japan) or a modified consumer-grade 20.1 MP Sony QX1 series camera (Sony Corporation, Minato, Tokyo, Japan). Both were converted to use NIR, green, and blue channels. On two flight dates in this study, a 20.1 MP Sony RX100 Mark III (Sony Corporation, Minato, Tokyo, Japan) capturing red, green, and blue bands was used (Table 2). Trends in data suggest that using a different sensor on the final flight had a negligible effect on the volume and groundcover estimations. The ground sample distance for each camera and altitude combination is shown in Table 2. The take-off and landing of the UAV were executed manually by the pilot, but the image acquisition portion of flights was performed utilizing a pre-programmed flight plan created with DraganFly Surveyor software. The Surveyor software automatically produces a flight plan with optimum speed and routing to obtain operator-prescribed parameters, including altitude, image overlap, and GSD. Images were collected from a nadir perspective with the payload saddle in the surveyor mode. Flight altitude was either 15 m or 20 m to allow relatively low GSD providing high-resolution imagery (Table 2). Image overlap was maintained at 70% or greater to enable orthomosaic and 3-D point cloud development. Ground control points with a known location measured using a Trimble 5800 model R8 real-time kinematic (RTK)-corrected GPS remained in the field throughout the season at all locations. The information from the ground control points was used in georectfication during image processing.

### 3.5. Image Processing

**Figure 8:**
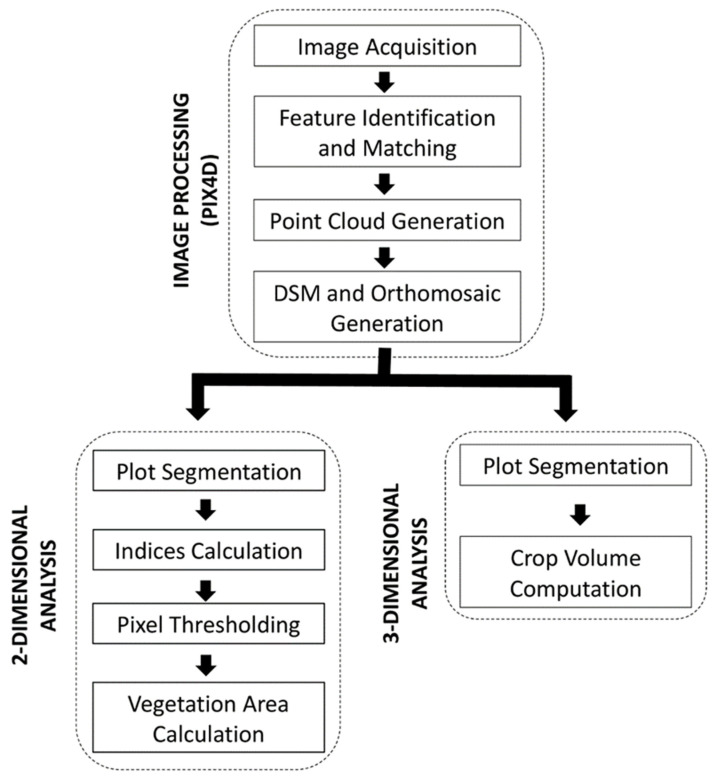
Image processing and analysis workflow.

The image processing was conducted using Pix4D software Version 4.3.1 (SA, Lausanne, Switzerland, 2018). Images acquired from the UAV platform were adjusted and calibrated for position/orientation, lens distortion, and other intrinsic parameters. Geo-rectification was performed using the coordinate information from the ground control points. Three-dimensional point clouds and stitched orthomosaics (Figure 7) were then produced for each location following the workflow shown in Figure 8.

### 3.6. Image Analysis: Two-Dimensional (2-D)

A 2-D analysis was used to determine the ground area covered by vegetation as viewed from a nadir perspective. ArcGIS software version 10.4.0.5524 [44] was used first for plot segmentation by manually creating bounding polygons on a 2-D orthomosaic around each plot, then applying indices to identify plant material from non-plant material (Figure 8). gNDVI (Green normalized difference vegetation index, Equation (2)) quite effectively identified green pixels while eliminating shadows and other non-green material. On some image dates, bNDVI (Blue normalized difference vegetation index, Equation (3)) was determined to be a better basis for green pixel identification based on visually observed indices characteristics. Only RGB imagery was available on biomass sample 6 at Sutherland 2017, so NGRDI (Normalized green, red difference index, Equation (4)) was used. Rasmussen et al. [45] suggested that using NGRDI (Normalized green red difference index) compared with NDVI (Normalized difference vegetation index) did not significantly inhibit the ability to assess green vegetation. Therefore, to quantify ground covered by green vegetation in this study, gNDVI, bNDVI, and NGRDI were considered to produce equivalent results as indices selection and threshold value selection were determined independently at each image analysis time-point. Once indices were calculated, thresholds were applied to eliminate all non-green pixels from the image.

(2) $$\mathrm{gNDVI}=\frac{\mathrm{NIR}-\mathrm{Green}}{\mathrm{NIR}+\mathrm{Green}}$$

(3) $$\mathrm{bNDVI}=\frac{\mathrm{NIR}-\mathrm{Blue}}{\mathrm{NIR}+\mathrm{Blue}}$$

(4) $$\mathrm{NGRDI}=\frac{\mathrm{Green}-\mathrm{Red}}{\mathrm{Green}+\mathrm{Red}}$$

Thresholds were determined by user-based visual inspection on each imaging date. By manually determining thresholds, the present study successfully separated vegetation from non-vegetative backgrounds such as soil and crop residue compared to the standardized threshold values (data not shown). Therefore, data for further analysis were derived from independently determined threshold values as this method. Green pixel count and calculated green pixel area were then determined for each plot using the raster calculator in ArcGIS based on pixels categorized as representative of vegetation. These values were then used in further analysis to negate variance between actual indices values.

### 3.7. Image Analysis: Three-Dimensional (3-D)

3-D dense point clouds were generated based on the sparse point cloud generated in the previous step of pix4d image processing. A textured mesh was constructed based on the dense point cloud, and the resulting 3-D model was used to calculate the 3-D volume of individual crop plots. The Pix4D volume measuring tool involves a calculation comparing the digital surface model (DSM) and digital terrain model (DTM). Volume information is extrapolated from DSM and DTM information within this manually specified area for each plot by applying a grid based on GSD spacing and determining the volume of each selected cell. The base height of the selected region is derived from the altitude of each user-selected vertex. Plot-bounding polygon vertices were determined manually on a plot-by-plot basis to contain the plot as precisely as possible using the total volume of the selected region as the sum of the volume of each cell within the selected area.

### 3.8. Statistical Analysis

Statistical analysis was performed using the DRC package [46] run in R [47] on RStudio version 1.1.456 [48]. Data for dry weight biomass, vegetation area, and plot volume at each site-year were truncated to remove late-season declining values. Three-parameter logistic non-linear models were constructed independently at each site-year (Figure 1, Figure 4 and Figure 6), with the initial value assumed to be zero. These models are frequently used in plant biomass modeling [49]. In the three-parameter model (Equation (5)), parameter *b* represents the growth rate around G50 or growth rate, parameter *d* represents the upper asymptote of the curve or predicted maximum growth, and *e* represents the G50 or the number of GDD required to accumulate 50% of maximum growth.

(5) $$f\left(x\right)=0+-\frac{d-0}{1+\exp\left(b\left(\log\left(x\right)-\log\left(e\right)\right)\right)}$$

Parameter estimation in the DRC package is based on the maximum likelihood principle. The “transform-both-sides approach” was executed using a Box-Cox transformation to control variance heterogeneity and help ensure a normal distribution [50,51].

For each site-year, genotypes were analyzed both individually and combined. An ANOVA was then performed, and all site-years except Nasser 2017 showed a significant overall difference between the model comparing individual genotypes and the model with genotypes combined (α = 0.05). Akaike Information Criterion (AIC) was compared among models at each site-year. AIC-based selection indicated that the model using individual genotypes explained greater variance in data at all site-years except Nasser 2017. Additionally, models for individual genotypes converged with insignificant lack-of-fit tests at all site-years, except Nasser 2017, indicating acceptable model fit. It was therefore determined that a model including individual genotypes should be used for Sutherland 2017, Rosthern 2017, Nasser 2018, and Rosthern 2018. Data from Nasser 2017 was best described by combing all genotypes within the model. To handle variance heterogeneity that may be present in the data, R functions *coeftest* and *sandwich* were used to obtain robust standard errors [50].

Package *corrplot* [52] running in R [28] using RStudio version 1.1.456 [47] was used to produce correlation matrices comparing dry weight biomass, green pixel area, and crop volume of each plot.

## 4. Conclusions

Overall, remotely detected vegetation area and crop volume were found to produce trends comparable to the accumulation of dry weight biomass throughout the growing season.

The maximum predicted volume was determined to be correlated with end-of-season biomass, and it is proposed that the evaluation of maximum predicted growth relative to other genotypes would be useful in estimating end-of-season plant biomass in research programs. The maximum predicted green pixel area might be used to quantify the time to canopy closure, which is a good indicator of early-season competitive potential [23]. The growth rate parameter of either dry weight biomass, green pixel area, or plot volume may be utilized to estimate plant growth rate. The fact that growth rate parameters have a reasonably high likeness when compared among these traits suggests that plant growth rate is similar among dry weight biomass, green pixel area, and plot volume. It is proposed that a growth rate calculated from sequential data collection events could be highly useful in a breeding program to evaluate germplasm for desirable growth rates either throughout the growing season or for the period of a particular physiological growth stage. Area G50 is also useful for quantifying the rate of canopy closure rate in 2-D data, and volume G50 are useful in quantifying the maximum volume growth rate in 3-D data.

The digitally measured vegetation area is useful for estimating lentil biomass and is indicative of leaf area early in the growing season. For mid to late-season biomass estimation, plot volume was determined to be a better estimator of plot biomass. These results coincide with observations made by Sun et al. [38]. By late-season data collection points, plot volume was either similarly or more highly correlated with dry weight biomass than vegetation area. Due to its consistent improvement in correlation with dry weight biomass throughout the growing season and high correlation with dry weight biomass late in the growing season, it is suggested that plot volume is an acceptable high-throughput proxy for dry weight biomass of mid to late-season analysis of lentil.

In the near future, image processing and analysis will likely be an automated or semi-automated process requiring minimal user inputs [53]. As large-scale continuous monitoring of plant parameters, including ones that are not typically collected due to resource limitations, is possible, authors believe that UAV image-based methods can create new opportunities to achieve improved breeding efficiency by affecting selection intensity.

## Figures and Tables

**Table 1:** Data collection at each location began two weeks after emergence and was repeated approximately every two weeks until all genotypes reached physiological maturity. Correlations were determined between dry weight (DW), vegetation area (Area), and plot volume (Volume) at each data collection time and site years.

	Data Collection 1			Data Collection 2			Data Collection 3			Data Collection 4			Data Collection 5			Data Collection 6		
**Nasser 2017**	**DW**			**DW**			**DW**			**DW**			**DW**					
	0.84	**Area**		0.97	**Area**		0.88	**Area**		0.82	**Area**		0.53	**Area**				
	0.20	0.19	**Volume**	0.81	0.83	**Volume**	0.86	0.86	**Volume**	0.80	0.98	**Volume**	0.85	0.70	**Volume**			
**Sutherland 2017**	**DW**			**DW**			**DW**			**DW**			**DW**			**DW**		
	0.90	**Area**		0.96	**Area**		0.90	**Area**		0.76	**Area**		0.99	**Area**		0.91	**Area**	
	0.36	0.28	**Volume**	0.85	0.88	**Volume**	0.94	0.97	**Volume**	0.91	0.79	**Volume**	1.00	0.98	**Volume**	0.97	0.92	**Volume**
**Nasser 2018**	**DW**			**DW**			**DW**			**DW**			**DW**			**DW**		
	0.87	**Area**		0.92	**Area**		0.90	**Area**		0.76	**Area**		0.96	**Area**		0.96	**Area**	
	0.17	0.40	**Volume**	0.42	0.43	**Volume**	0.69	0.68	**Volume**	0.83	0.81	**Volume**	0.77	0.75	**Volume**	0.95	0.91	**Volume**
**Rosthern 2017**	**DW**			**DW**			**DW**			**DW**			**DW**			**DW**		
	0.84	**Area**		0.90	**Area**		0.93	**Area**		0.82	**Area**		0.77	**Area**		0.96	**Area**	
	−0.03	−0.26	**Volume**	0.85	0.74	**Volume**	0.77	0.88	**Volume**	0.57	0.74	**Volume**	0.34	0.62	**Volume**	0.62	0.60	**Volume**
**Rosthern 2018**	**DW**			**DW**			**DW**			**DW**			**DW**			**DW**		
	−0.30	**Area**		0.95	**Area**		0.89	**Area**		0.68	**Area**		0.89	**Area**		0.92	**Area**	
	0.02	0.08	**Volume**	0.43	0.44	**Volume**	0.63	0.78	**Volume**	0.64	0.95	**Volume**	0.68	0.79	**Volume**	0.78	0.77	**Volume**

**Table 2:** Ground Sample Distance (GSD) of each camera/altitude combination used throughout the experiment. GSD is the distance between two consecutive pixel centers measured on the ground, it is a measure of camera spatial resolution.

Camera (Lens, Focal Length, F-Stop)	Altitude (m)	Ground Sample Distance (mm)
Sony RX100 Mark III 20.1 MP	15	4.1
(24–70 mm, 8.8 mm, f/1.8–2.8)	20	5.5
Sony QX1 20.1 MP	15	5.3
(16 mm, 24 mm, f/2.8)	20	4
Sony α5100 24.3 MP	15	4.9
(16 mm, 24 mm, f/2.8 mm)	20	7

## Data Availability

Not applicable.
